# Central Role of Cell Cycle Regulation in the Antitumoral Action of Ocoxin

**DOI:** 10.3390/nu11051068

**Published:** 2019-05-14

**Authors:** Javier Pérez-Peña, Elena Díaz-Rodríguez, Eduardo Sanz, Atanasio Pandiella

**Affiliations:** 1 Instituto de Biología Molecular y Celular del Cáncer, CSIC and CIBERONC, 37007 Salamanca, Spain; javippv@gmail.com (J.P.-P.); ediaz@usal.es (E.D.-R.); 2 Centro Regional de Investigaciones Biomédicas, Universidad de Castilla La Mancha, 02008 Albacete, Spain; 3 Research and Development, Catalysis S.L., 28016 Madrid, Spain; eduardo@catalysis.es

**Keywords:** small-cell lung cancer, acute myeloid leukemia, antioxidants, cell cycle, p27, apoptosis

## Abstract

Nutritional supplements which include natural antitumoral compounds could represent safe and efficient additives for cancer patients. One such nutritional supplement, Ocoxin Oral solution (OOS), is a composite formulation that contains several antioxidants and exhibits antitumoral properties in several in vitro and in vivo tumor conditions. Here, we performed a functional genomic analysis to uncover the mechanism of the antitumoral action of OOS. Using in vivo models of acute myelogenous leukemia (AML, HEL cells, representative of a liquid tumor) and small-cell lung cancer (GLC-8, representative of a solid tumor), we showed that OOS treatment altered the transcriptome of xenografted tumors created by subcutaneously implanting these cells. Functional transcriptomic studies pointed to a cell cycle deregulation after OOS treatment. The main pathway responsible for this deregulation was the E2F–TFDP route, which was affected at different points. The alterations ultimately led to a decrease in pathway activation. Moreover, when OOS-deregulated genes in the AML context were analyzed in patient samples, a clear correlation with their levels and prognosis was observed. Together, these data led us to suggest that the antitumoral effect of OOS is due to blockade of cell cycle progression mainly caused by the action of OOS on the E2F–TFDP pathway.

## 1. Introduction

From the beginning of medical history, natural compounds were used in the treatment of different diseases [1,2]. Thus, in the oncology field, vinca alkaloids or taxanes were identified as antitumoral drugs and used to treat patients [3,4,5,6]. Based on those active principles, more efficient drugs have been developed and have extensively been used in the oncology clinic [7]. Nonetheless, the use of agents in monotherapy is a rare event in the clinic, and combinations of therapeutic agents are very commonly employed in oncology. On the basis of this concept, several therapeutic formulations, consisting in combinations of natural products with demonstrated antitumoral capabilities have been developed. One of the advantages of such natural product combinations is their high therapeutic index, allowing their safe use. 

In the last several years, several independent studies have demonstrated the antitumoral action of one such product, termed Ocoxin [8,9], either alone or in combination with other drugs commonly used in the oncology clinic. This product is given orally (Ocoxin Oral Solution, OOS, Catalysis, Villaluenga de la Sagra, Toledo, Spain), and its formulation includes the green tea polyphenol epigallocathechin, vitamin B, vitamin C, and cinnamic acid, all of them with demonstrated antitumor activity [10,11,12,13]. In addition, OOS includes other components with anti-inflammatory and immunomodulatory potential such as glycyrrhizinic acid [14]. In breast cancer cells, OOS has shown capability to reduce tumor cell proliferation and increase the antitumoral action of lapatinib, which is used to treat HER2 positive (HER2+) breast cancer [15]. Similarly, OOS has been shown to reduce the proliferation of colon cancer cells and to also reduce the metastatic spreading of tumor cells [16,17]. In addition, OOS has demonstrated antitumoral capability in in vitro and in vivo models of hepatocellular carcinoma [18], small-cell lung cancer [19], and acute myeloblastic leukemia [20]. Mechanistically, the antitumoral action of OOS has been explored using in vitro models. In those studies, OOS demonstrated capability to affect the cell cycle, inducing an extension in the G1 phase of the cell cycle, likely accounting for its antitumoral effect. In addition, a certain effect of OOS on the induction of apoptosis was observed. The effect of OOS on the cell cycle was accompanied by an increase in the expression of the negative regulator of cell cycle progression p27 [15,18,19,20]. To gain additional insights about the mechanism of the antitumoral action of OOS, we performed a functional transcriptomic analysis of xenografted tumors from mice treated with OOS. These studies confirmed that the major antitumoral action of OOS is due to an effect on cell cycle progression and define the retinoblastoma route as an important pathway in the action of OOS.

## 2. Material and Methods

### 2.1. OOS Composition

OOS is a nutritional supplement which has been licensed for use in more than 100 countries. OOS formulation includes per 50 mL of solution: L-glycine 1000 mg, glucosamine sulfate 1000 mg, L-arginine 320 mg, L-cysteine 102 mg, licorice (*Glycyrrhiza glabra*) 100 mg, vitamin C (L-ascorbic acid) 60 mg, water, zinc sulfate 40 mg, green tea extract (*Camellia sinensis*) 12.5 mg, vitamin B5 6 mg, vitamin B6 2 mg, manganese sulfate 2 mg, cinnamon extract (*Cinnamomum zeylanicum*) 1.5 mg, folic acid 200 µg, vitamin B12 1 µg, acidulant (malic acid), and preservative (sodium methylparaben). 

### 2.2. Cells Culture and Animal Experiments

A model cell line for each experimental condition was chosen and grown in RPMI-1640 medium supplemented with 10% fetal bovine serum and antibiotics. The model cell line used for small-cell lung cancer (SCLC) was GLC8, while for acute myeloblastic leukemia (AML), it was the HEL cell line. Cells were obtained as described [19,20] and cultured in standard conditions, at 37 °C in a humidified atmosphere in the presence of 5% CO_2_.

For animal studies, seven-week-old female athymic mice (CB17-SCID) were purchased from Charles River Laboratories and maintained in pathogen-free housing at our Institutional Animal Care Facility. Animal experiments were performed according to the institutional guidelines and protocol approved by the Ethics Committee of the University of Salamanca. One week later, 6 × 10^6^ GLC8 cells or 4 × 10^6^ HEL cells were resuspended in 50 μL of RPMI medium mixed with 50 μl Matrigel (BD Biosciences, San Jose, CA, USA) and subcutaneously injected into the right caudal flank of each animal. When the tumors were palpable, the animals were randomized into two groups, including six animals per group. One group of animals was treated with vehicle alone (water), while the other group of mice were orally treated with 100 mL OOS per animal. Such dose of OOS has been reported to be effective in previous studies [18,20]. Treatments were carried out with a daily schedule (Monday to Friday), and tumor growth was measured twice a week with a digital caliper. Tumor volumes were calculated with the formula: V = (L/2) × (W/2)^2^ × 4/3 × π, where V = volume (mm^3^), L = length (mm) and W = width (mm). After 31 (GLC8) or 17 (HEL) days of treatment, tumors were resected and immediately frozen at −80 °C.

### 2.3. Functional Genomic and Transcriptomic Profiling

RNA from three tumors for each experimental condition was isolated using Trizol (Life Technologies, Carlsbad, CA, USA) following the manufacturer´s instructions. The tumors analyzed belonged to mice in which the in vivo effect of OOS on the growth of such tumors was already published by our group [19,20]. The extracted RNA was quantified and hybridized to HuGene 2.0 GeneChip (AML, HEL cells) or to Clariom S Human (SCLC, GLC8 cells) oligonucleotide arrays (Affymetrix, Santa Clara, CA, USA) and scanned as described [19,20]. Unprocessed CEL files were normalized separately (AML and SCLC) using the RMA algorithm implemented in the Affymetrix Expression Console 4 (Santa Clara, CA, USA). Generated CHP processed files were then analyzed using Transcriptome Analysis Console (TAC, Affymetrix), establishing a cut-off for differentially expressed genes (DEGs) of ≥2-fold change and a *p* value ≤0.05. 

Gene set enrichment analyses (GSEA, Broad Institute Inc., MIT, MA, USA) were performed to identify gene sets classified into five different cellular functions (Cell Cycle, Cell Death, Immune Response, Cell Differentiation, and Transcription) showing expression alterations between control and OOS-treated tumors [21,22]. In total, 206 gene sets were collected from the Molecular Signatures Database (MSigDB) (http://www.broadinstitute.org/gsea/msigdb/); the data were analyzed by GSEA with parameter set to 1000 gene-set permutations. This analysis yielded an enrichment score that, when positive (e.g., the gene set was overrepresented by top-ranked genes), indicated that the gene set was upregulated. On the other side, the gene set was considered downregulated when the score was negative. A network of gene sets interactions was constructed by using the Cytoscape software (version 3.4.0, Institute of Systems Biology, Seattle, WA, USA). Normalized Enrichment Score (NES) values were normalized with respect to the number of genes that composed them. The means of those values obtained after normalization of every gene set were considered the Average Normalized Enrichment Score (Avg NES) of each sub-classification (Cell Cycle, Cell Death, Immune Response, Cell Differentiation and Transcription). 

Microarray data from Clariom S Human (SCLC, GLC8 cells) oligonucleotide arrays will be available through the GEO repository database.

### 2.4. Evaluation of Transcription Factor Association with Deregulated Genes

Association significance between deregulated genes and transcription factors was validated using the Max Planck Institute online tool PASTAA (http://trap.molgen.mpg.de/cgi-bin/pastaa.cgi), which uses a physical model to predict the relative binding affinities of transcription factors to regulatory regions of the DNA responsible for the transcription of provided genes [23].

### 2.5. E2F Pathway Map Development and Activation Score

The E2F pathway was collected from the Pathway Interaction Database (PID), via the NDEx database (www.ndexbio.org) and represented through the Cytoscape software with data from TAC analyses. PTGDR/PTGDR2, SMARCA2, MAPK1, and CDC25A were considered positive regulators of E2F pathway activation, while E2F8, PRMT5, CDKN2A, and CDKN2C were considered negative regulators [24]. Signal (log2) expression values from each replicate (Control and OOS treated) were normalized against their respective controls (1.Control1_(HuGene-2_0-st) in AML and 1.C1_(Clariom_S_Human) in SCLS). These normalized expression values of the genes defined as positive regulators of E2F pathway activation were added, while the normalized expression values of those genes defined as negative regulators (that blocked E2F1–TFDP1 activation) were deleted. The value obtained was considered the E2F pathway activation score.

### 2.6. TCGA Patients Gene Expression Comparison and Outcome Analyses

The Firebrowse online tool (http://firebrowse.org/) was used to compare the expression of deregulated genes in each different TCGA gathered tumor types. 

The PROGgeneV2 Online Tool (http://genomics.jefferson.edu/proggene/) was used to evaluate the relationship among the deregulated genes and TCGA patient relapse-free survival in AML. The median threshold between low and high expression was used as a cutoff.

### 2.7. Statistical Analyses

The value of *p* < 0.05 was considered to indicate a statistically significant difference.

## 3. Results

### 3.1. Genes Deregulated by OOS In Vivo

**Figure 1:**
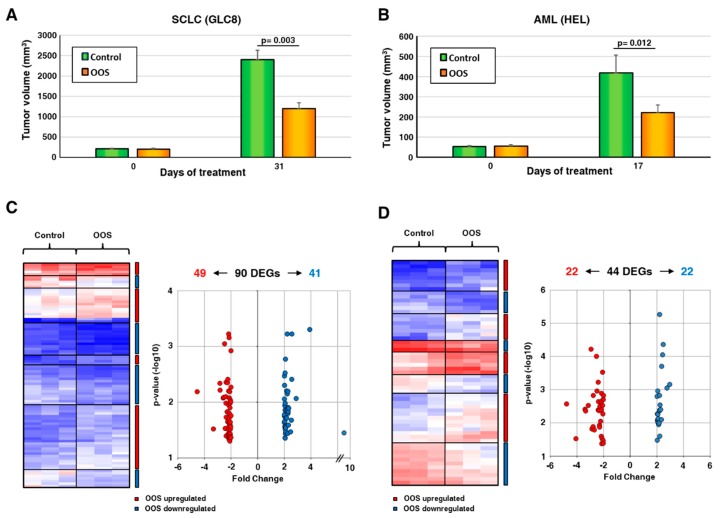
Ocoxin Oral Solution (OOS) transcriptomic modulation. (**A**). In vivo effect of OOS on the growth of small-cell lung cancer (SCLC) tumor models. Animals were injected with GLC8 cells and, when the tumors were correctly engrafted and growing, were randomized into two groups that were daily treated with PBS (control, green) or OOS (orange). Tumor volumes at the end of the treatment were calculated. Data are represented as mean tumor volume ± SD, and statistical differences are indicated. (**B**) In vivo effect of OOS on tumor growth of acute myeloblastic leukemia (AML) models. Similarly, animals were injected with HEL cells, and tumor growth was followed after OOS daily treatment. (**C**) RNA from three different SCLC tumors for each condition was prepared and analyzed by Affymetrix arrays, as described. Differentially Expressed Genes (DEG) with a minimum of two-fold change differential expression and a maximum 0.05 *p* value after OOS treatment are shown. Overexpressed genes are displayed in shades of pink-red, and downregulated genes in shades of blue. (**D**) A similar approach was used to analyze AML-derived tumors.

To evaluate the transcriptomic effects of OOS, we used two different in vivo models, based on the injection of acute myeloid leukemia HEL cells or small-cell lung cancer GLC8 cells in mice. We selected those cell lines since former studies have shown that they are sensitive to the antitumoral action of OOS [19,20]. Analyses of data generated in our laboratory [19,20] showed that tumors from the GLC8 cell line grew faster to higher volumes than those from HEL cells (Figure 1A,B and references [19,20]). When tumors were palpable and started to grow, the animals were randomly separated in two groups, and one group was treated with OOS. After four weeks of treatment, OOS significantly reduced tumor growth (Figure 1A,B). In fact, in the SCLC model, the mean tumor volume of the control tumors was 2402.6 ± 223.6 mm^3^, and after 31 days on OOS diet, the tumors reached a volume of 1197 ± 144.4 mm^3^ (*p* = 0.003, Figure 1A, see also reference [19]). As for the AML model, control tumors grew up to 484 ± 75.8 mm^3^, while OOS-treated tumors grew up to 222 ± 37.3 mm^3^, with *p* = 0.012 (Figure 1B, see also reference [20]). Tumors were then resected, and RNA was extracted from tumor regions free of necrosis. The extracted RNA was then subjected to microarray analyses. 

Transcriptomic studies were carried out in parallel in both models, and genes with a minimum two-fold change differential expression and at least 0.05 *p* value were selected. These studies defined the presence of 90 differentially expressed genes (DEGs) in the SCLC model (Figure 1C). Of them, 49 were downregulated by OOS, while 41 were upregulated. In AML tumors, treatment with OOS provoked the deregulation of 44 DEGs (Figure 1D), of which 22 were downregulated and 22 were upregulated. The accompanying supplementary Table S1 details the deregulated genes found in both in vivo models as well as the magnitude of their deregulation, indicated by the fold-change values. 

### 3.2. Functional Transcriptomic Analyses in AML and SCLC Tumors

**Figure 2:**
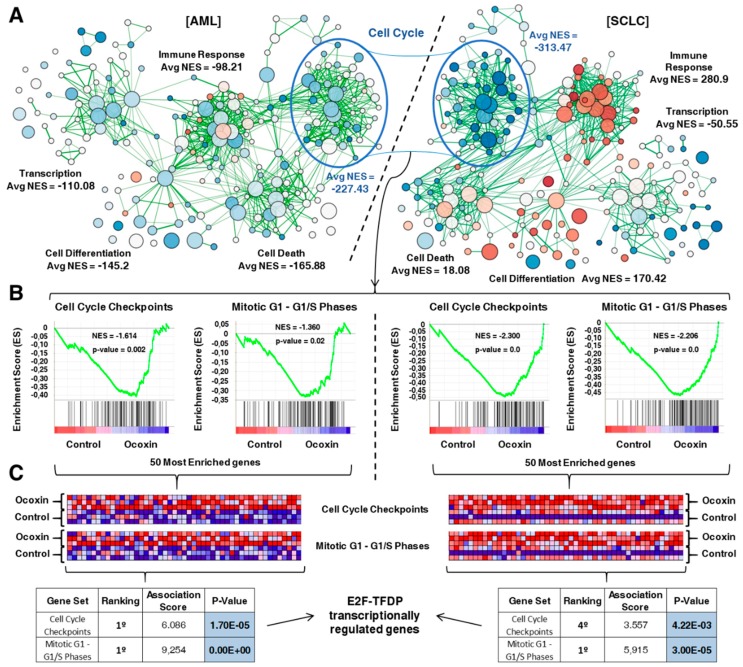
Functional analysis of transcriptomic alterations caused by OOS. (**A**). Gene-set enrichment expression network designed using Cytoscape to compare control and OOS-treated cells in our two experimental models, AML (left) and SCLC (right). Several cellular functions were clearly deregulated by OOS. Cell Cycle had the highest average normalized enrichement score (Avg NES) in both tumor models. (**B**) Enrichment score (ES) profile and location of GeneSet members on the rank-ordered list of the two most deregulated cell cycle GeneSets (“Cell Cycle Checkpoints” and “Mitotic G1-G1/S Phases”) in the AML model (left panels). Both ES profiles behaved very similarly in the SCLC model (right panels). (**C**) Blue-pink diagram of the 50 most OOS-deregulated genes included in “Cell Cycle Checkpoints” and “Mitotic G1-G1/S Phases”. Overexpressed genes are displayed in shades of pink-red, and downregulated genes in shades of blue. PASTAA online tool results are displayed in the table. The highest association score for three of the four pools of genes analyzed correlated the expression of these genes to the E2F pathway.

To gain insights into the functional consequences associated with the transcriptional alterations observed upon treatment of mice with OOS, gene-set enrichment analyses were performed in both models. These studies generated two different transcriptional maps after analysis using the Cytoscape software (Figure 2A). These analyses pointed to the deregulation of several cellular functions after OOS treatment, such as immune response, cell death, cell differentiation, and transcription. Of note, normalized enrichment scores, which are a measure of the strength of pathway/function deregulation, indicated that the cell cycle was the most altered function, presenting a similar profile of alteration in both AML and SCLC in vivo models. Immune response functions were also highly deregulated in SCLC, and functions related to cell death were substantially affected in AML.

Within the cell cycle category of deregulated genes, those corresponding to subcategories related to “Cell Cycle Checkpoints” and “Mitotic G1–G1/S Phases” were the most deregulated GeneSets in AML (Figure 2B, left panels), ranking 1st and 2nd, respectively. These subcategories were also deregulated in the SCLC model (Figure 2B, right panels), ranking also 1st in the case of “Cell Cycle Checkpoints” and 3rd in the case of “Mitotic G1–G1/S Phases”. Supplementary Table S2 shows those GeneSets within the cell cycle category that were significantly deregulated in both in vivo models as well as the magnitude of their deregulation, indicated by the NES. 

To gain additional insights into the action of OOS on the cell cycle, the 50 most overexpressed genes which belong to each of the “Cell Cycle Checkpoints” and “Mitotic G1–G1/S Phases” GeneSets were evaluated in both tumor models using the Max Planck Institute online tool PASTAA. These analyses offered an association score that related the examined genes to those factors most likely to be involved in their transcription. In this case, the analyses indicated that the E2F–TFDP transcriptional pathway was the most deregulated route (highest association score) in three of the four GeneSets explored (Figure 2C). These results pointed to the implication of the E2F–TFDP route in the effect of OOS. 

### 3.3. Action of OOS on the E2F–TFDP Transcriptional Network 

**Figure 3:**
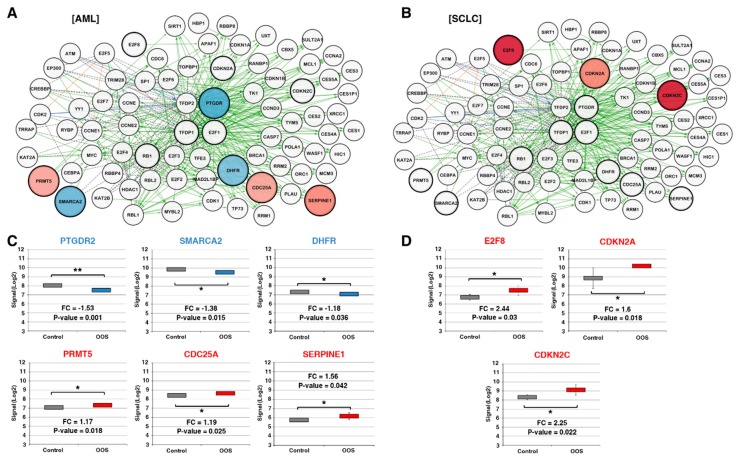
Components of the E2F transcriptional network are deregulated after OOS treatment. (**A**). Cytoscape software representation of the E2F transcriptional network, composed of a total of 80 interactors in the AML model. Overexpressed genes are displayed in shades of pink-red, and downregulated genes in shades of blue; the color intensity is proportional to the fold-change values. Black dotted labels define “in complex with” interactions, green solid labels define “controls expression of” interactions, blue labels define “controls state change of” interactions, and orange labels “controls phosphorylation of” interactions. (**B**) Similarly, E2F transcription network is shown in SCLC. (**C**) Statistically significant deregulated E2F pathway genes in AML or in SCLC (**D**). Names in red indicate overexpression after OOS treatment, while those in blue are indicative of OOS downregulation. * indicate statistically significant differences.

The main function of the E2F–TFDP transcriptional network is the regulation of the G1-to-S checkpoint [25,26,27]. According to the Pathway Interaction Database online tool, the E2F transcription network is composed of a total of 80 interactors (Figure 3A,B). The effect of OOS on each individual gene was analyzed using the Transcriptome Analysis Console which utilizes microarray expression data. Expression values were then represented through the Cytoscape software in the AML (Figure 3A) and SCLC models (Figure 3B). In AML tumors, these analyses showed that OOS caused the upregulation of CDC25A (fold change: 1.19, *p*-value: 0.026), PRTM5 (fold change: 1.17, *p*-value: 0.018), and SERPINE1 (fold change: 1.56, *p*-value: 0.042), and the downregulation of SMARCA2 (fold change: −1.38, *p*-value: 0.015), PTGDR2 (fold change: −1.53, *p*-value: 0.001), and DHFR (fold change: −1.18, *p*-value: 0.036) (Figure 3A,C). On the other hand, these studies showed that OOS provoked the up-regulation of E2F8 (fold change: 2.44, *p*-value: 0.03), CDKN2A (fold change: 1.6, *p*-value: 0.018), and CDKN2C (fold change: 2.25, *p*-value: 0.022) in SCLC tumors (Figure 3B, D). 

**Figure 4:**
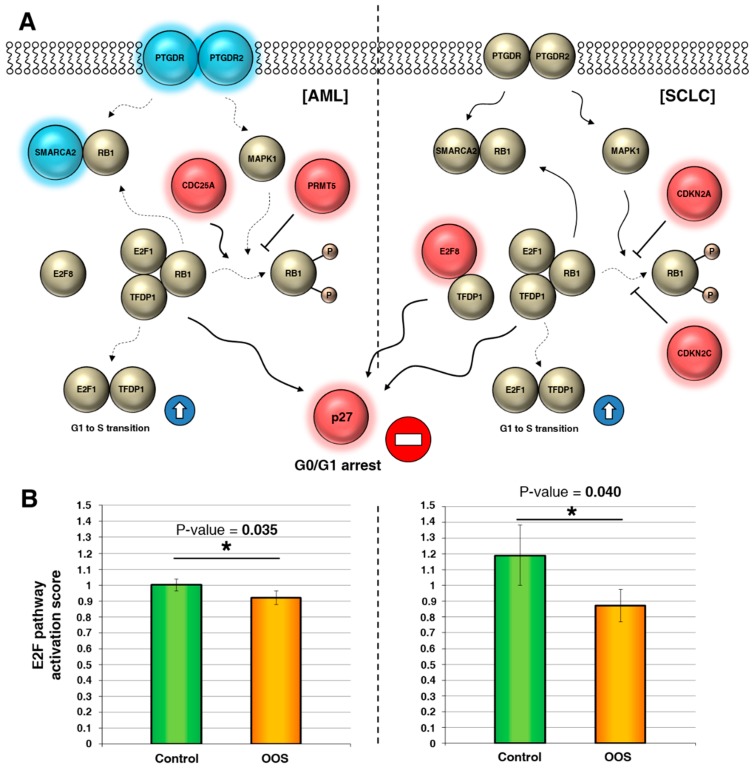
OOS downregulates the E2F pathway. (**A**) Interactions among E2F pathway genes deregulated by OOS in AML (left) or SCLC (right), represented in a flow chart network. Overexpressed genes after OOS treatment are displayed in shades of pink-red, and downregulated genes in shades of blue. (**B**) OOS contributes significantly to block the activation of the E2F1–TFDP1 pathway mediated by its regulators in both tumor models. E2F pathway activation scores were estimated as described in the Material and Methods section. * indicate statistically significant differences.

The altered genes identified in the above study participate in the regulation of the E2F pathway by acting at several levels (Figure 4A). Genes represented in blue were downregulated, while the red genes were upregulated by OOS. The genes downregulated by OOS were positive regulators of the pathway. Conversely, upregulated genes upon OOS treatment were negative regulators. An E2F pathway activation score was calculated as indicated in the materials and methods section. In those conditions, the statistical analyses of the effect of these deregulated genes indicated that treatment with OOS negatively affected E2F pathway activity (Figure 4B) both in AML and in SCLC.

### 3.4. Prognostic Relevance of the E2F–TFDP Route

**Figure 5:**
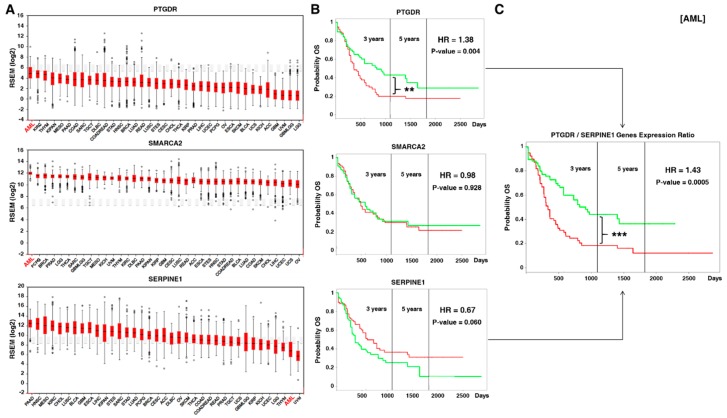
E2F–TFDP route has prognostic relevance in AML patients. (**A**) Within OOS-deregulated genes, two of them (PTGDR and SMARCA2) had the highest expression in AML compared to other tumor types, while SERPINE1 had the second lowest expression (data from TCGA patients). (**B**) Prognostic relevance of the expression of PTGDR, SMARCA2, and SERPINE1 individually in AML patients. PTGDR expression is significantly related with worse relapse-free survival in AML patients. The green line corresponds to patients with low amounts of the corresponding protein, while the red line indicates higher levels. (**C**) The PTGDR/SERPINE1 expression ratio significantly correlates with worse relapse-free survival in AML patients. The combination of high expression of PTGDR and low expression of SERPINE1 is depicted in red, while the combination of low PTGDR expression and high SERPINE1 expression is shown in green. ** and *** indicate statistically significant differences.

With the aim of evaluating whether this pathway has also relevance in a clinical context, we explored the expression of the deregulated genes which participate in the E2F pathway in TCGA (The Cancer Genome Atlas) samples, comparing their expression among different tumors with available data. For those genes deregulated after OOS treatment, PTGDR and SMARCA2 turned out to be more expressed in AML than in any other tumor type, conversely, SERPINE1 was less expressed in AML than in most other tumor types (Figure 5A).

We then explored the prognostic relevance of these genes in the AML context. PTGDR (HR, hazard ratio: 1.38, *p*-value: 0.004) significantly correlated with overall survival in AML patients (Figure 5B). On the other hand, SMARCA2 (HR: 0.98, *p*-value: 0.928) did not show any correlation with patient outcome. SERPINE1 (HR: 0.67, *p*-value: 0.060) showed a trend to better prognosis if upregulated (Figure 5B). When considering the expression ratio PTGDR/SERPINE1, the prognostic relevance was even better (HR: 1.43, *p*-value: 5x10E-4) (Figure 5C). 

## 4. Discussion

OOS is a nutritional supplement which contains several natural products, some of them with proven antioxidant and antitumoral properties [10,11,12,14,28,29,30]. Because of the characteristics of such products, including their ample therapeutic index, their use is safe and may offer therapeutic value associated with the antitumoral properties of several of its constituting compounds. It is therefore relevant to uncover how OOS acts on tumor cells to achieve such benefit. 

Former studies indicated that OOS decreased tumor burden in vivo [15,18,19,20]. The action of a product that decreases tumor volume may be caused by an increase in cell death, a decrease in the progression of the tumor cells along the cell cycle, or a combination of these mechanisms. In vitro studies carried out in a number of different cell lines representative of distinct tumors showed that OOS slowed the progression of the cells along the cell cycle. In addition, OOS was shown to induce the activation of caspases and processing of PARP, which are indicative of the stimulation of apoptotic cell death [31,32,33,34]. With respect to the action of OOS on the cell cycle, former biochemical analyses demonstrated that OOS provoked an increase in the amount of p27 in several of the tumor models explored, both in vitro and in vivo [15,19,20]. That finding was compatible with a blockade of the cells in the G1 phase of the cell cycle. Here, by using a transcriptomic approach, we gained insights into the mechanism of the antitumoral action of OOS. The study used tumor material obtained from animals treated with OOS and bearing tumor cells of two types, the small-cell lung cancer cell line GLC8 and the HEL acute myeloblastic leukemia cell line. Xenografted cells from these two tumor types generated tumors whose growth was inhibited by OOS. Functional analyses of the expression data in tumors from mice treated with OOS revealed the deregulation of genes involved in several functions, including transcriptional regulation, immune responses, cell differentiation, or cell death. However, the mostly deregulated function in both tumor models treated with OOS was the cell cycle. This finding falls in line with previously published observations that implicate cell cycle progression in mediating the antitumoral action of OOS [15,18,19,20]. 

Within cell cycle regulation, a group of genes were identified as particularly deregulated: those corresponding to cell cycle checkpoints. Functional analyses of genes involved in cell cycle checkpoints finally led to the identification of the E2F–TFDP pathway as the major deregulated route in the action of OOS. The E2F transcription factors act on promoters of several genes that are important for cell cycle progression [35,36]. E2F interaction with critical regulatory proteins such as the retinoblastoma protein, modulates the transcriptional properties of E2F [25,37,38]. The functional transcriptomic analyses identified nine E2F/TFDP genes deregulated by OOS. Additional studies on the expression and importance of those genes in human tumors showed that AML had the highest levels of two of them, PTGDR and SMARCA2. In addition, AML was the second tumor with lower levels of SERPINE1. Additional in silico studies using data from the Firebrowse and TCGA allowed analyses of the potential correlation between the expression of these genes and patient outcome. Interestingly, increased levels of PTGDR negatively correlated with patient survival. On the other hand, higher levels of SERPINE1 favored a better patient outcome. The analysis of the prognostic relevance of these genes together was even more potent in terms of overall survival. Therefore, the action of OOS in reducing the expression of PTGDR together with the effect of OOS in increasing SERPINE1 may result in clinical benefit. These data are relevant as point to AML as a sensitive disease in which OOS may exert a significant transcriptional regulation of genes participating in cell cycle progression in malignant AML cells.

Together, the data we present here reinforce previous results indicating that OOS antitumoral action depended on an effect of the compound on the cell cycle [15,18,19,20]. The studies reported here extend previous mechanistic studies to indicate that one of the major functions deregulated by OOS is that affecting transcription by the E2F pathway, corroborating some recent data pointing to the role of one of OOS components (green tea extract) in regulating that pathway [39]. Besides, one of the components of licorice extract, glycyrrhetinic acid, has been implicated in the regulation of G1–S transition though the regulation of E2F factors in lung cancer [40]. As such route is implicated in the control of the expression of proteins important for cell cycle progression, our findings support that the mechanism of action of OOS clearly involves the negative regulation of such cellular function. Identification of oncologic diseases in which such function is more vulnerable to attack to reduce the proliferation of tumor cells may favor the personalization of the use of OOS to optimize its antitumoral properties. Studies in that direction should be performed.

## Supplementary Materials

The following are available online at https://www.mdpi.com/2072-6643/11/5/1068/s1, Table S1: Genes in vivo deregulated by OOS in the small cell lung cancer or the acute myeloid leukemia models. Table S2: GeneSets within the cell cycle category significantly deregulated in either the small cell lung cancer or acute myeloid leukemia in vivo models.
