# Supplementary material for: Central Role of Cell Cycle Regulation in the Antitumoral Action of Ocoxin
Source: Nutrients. 2019 May 14;11(5):1068. doi: 10.3390/nu11051068 (PMC6566638; doi:10.3390/nu11051068)
Supplement: Supplementary file 1 [file nutrients-11-01068-s001.pdf]

Para obtener la mejor experiencia, abra esta cartera PDF en Acrobat X o Adobe Reader X, o en alguna versión posterior.

¡Consiga Adobe Reader ahora!
